# Machine learning perioperative applications in visceral surgery: a narrative review

**DOI:** 10.3389/fsurg.2024.1493779

**Published:** 2024-10-30

**Authors:** Intekhab Hossain, Amin Madani, Simon Laplante

**Affiliations:** ^1^ Department of Surgery, University of Toronto, Toronto, ON, Canada; ^2^ Surgical Artiﬁcial Intelligence Research Academy, University Health Network, Toronto, ON, Canada; ^3^ Department of Surgery, Mayo Clinic, Rochester, MN, United States

**Keywords:** machine learning (ML), preoperative, intraoperative, postoperative, applications

## Abstract

Artificial intelligence in surgery has seen an expansive rise in research and clinical implementation in recent years, with many of the models being driven by machine learning. In the preoperative setting, machine learning models have been utilized to guide indications for surgery, appropriate timing of operations, calculation of risks and prognostication, along with improving estimations of time and resources required for surgeries. Intraoperative applications that have been demonstrated are visual annotations of the surgical field, automated classification of surgical phases and prediction of intraoperative patient decompensation. Postoperative applications have been studied the most, with most efforts put towards prediction of postoperative complications, recurrence patterns of malignancy, enhanced surgical education and assessment of surgical skill. Challenges to implementation of these models in clinical practice include the need for more quantity and quality of standardized data to improve model performance, sufficient resources and infrastructure to train and use machine learning, along with addressing ethical and patient acceptance considerations.

## Introduction

There has been rapid growth of interest over the past decade in the use of artificial intelligence (AI) in the field of surgery to perform data-driven tasks efficiently and ultimately improve patient care (1). Machine learning (ML) is a division of AI which learns from large datasets and algorithms to provide personalized analysis and predictions. In visceral surgery, applications of ML in surgery include optimization of patients and resources, intraoperative analysis and feedback, and prediction of postoperative complications.

Despite the increase in research and demonstration of application on ML in visceral surgery, there remains a challenge in implementation. Numerous factors play a role in this dilemma including ML training, validating and testing of quality data, model selection, implementation of appropriate resources and infrastructure for ML, along with ethical and professional acceptance (2).

In this mini review, we will assess current literature on application of ML in visceral surgery in the preoperative, intraoperative, and postoperative settings (Table 1).

**Table 1:** Summary of studies on application of machine learning in visceral surgery in the preoperative, intraoperative, and postoperative settings.

Setting	Authors, Year	Study design	Data source	Aim of ML model	Results of ML models
Preoperative	Henn et al. 2022 (3)	Systematic review	47 studies	Guide clinical decision-making to proceed with abdominal surgery	ML models were superior in 97.8% of studies vs. conventional methods
	Chongo et al. 2024 (4)	Systematic review	23 studies	Liver transplantation prognostication	ML models had excellent predictive performance (AUROC 0.9-1) for short and long term outcomes of mortality and post-transplantation complications
	Zhao et al. 2019 (5)	Retrospective cohort	Single institution, *n* = 500	Predict case duration during robot assisted surgery	ML modes were more accurate (51.7%) vs. conventional means (34.9%)
	Li et al. 2024 (6)	Retrospective cohort	Single institution, *n* = 186	Predict surgical difficulty of laparoscopic rectal procedures	ML models had high performance (AUROC 0.9-1) for predicting surgical difficulty
	Cao et al, 2019 (7)	Retrospective cohort	National registry, *n* = 37,811	Predict severe postoperative bariatric surgery outcomes based on preoperative factors	Most ML models had high accuracy (>90%) and specificity (>90%) but sensitivity (0–75%) and AUROC (around 0.5) were poor
	Lan et al. 2020 (8)	Retrospective cohort	Single institution, *n* = 223	Predict appropriate surgical timing for infected necrotizing pancreatitis	ML models better predicted surgical timing, identified factors associated with surgical timing, and postoperative survival for infected necrotizing pancreatitis vs. conventional methods
Intraoperative	Laplante et al. 2023 (9)	Retrospective cohort	International database, *n* = 31	Validate Madani et al's “GoNoGoNet” model of displaying safe and dangerous zones during laparoscopic cholecystectomy (10)	Model had 92% mean accuracy, 97% specificity, and 70% positive predictive value for safe zones and 92% mean accuracy, 80% sensitivity, and 95% negative predictive value for dangerous zones
	Mascagni et al. 2022 (11)	Retrospective cohort	Single institution, *n* = 201	“DeepCVS” model to identify critical view of safety in laparoscopic cholecystectomy	Model had mean accuracy of 71.4%
	Aspart et al. 2022 (12)	Retrospective cohort	Single institution, *n* = 300	“ClipAssistNet” model to warn about adequate clipper tip visibility prior to clipping cystic structures during laparoscopic cholecystectomy	Model had AUROC of 0.9107, 66.15% speciﬁcity and 95% sensitivity
	Garrow et al. 2021 (13)	Systematic review	35 studies	Automated surgical phase recognition during laparoscopic cholecystectomy, sleeve gastrectomy, colorectal surgery amongst others	ML models had accuracy ranging from 68.8% to 96.3%
	Takeuchi et al. 2022 (14)	Retrospective cohort	Single institution, *n* = 119	Automated surgical phase recognition during laparoscopic inguinal hernia repair	Models had accuracy of 88.1% and 85.8% in unilateral and bilateral repairs, respectively
	Kitaguchi et al. 2020 (15)	Retrospective cohort	Single institution, *n* = 71	Automated surgical phase recognition during laparoscopic sigmoidectomy	Model had accuracy of 91.9%
	Kitaguchi et al. 2022 (16)	Retrospective cohort	Single institution, *n* = 50	Automated surgical phase recognition during transanal total mesorectal excision	Model had accuracy of 93.2%
	Cheng et al. 2022 (17)	Retrospective cohort	Multicentre, *n* = 90	Automated surgical phase recognition during laparoscopic cholecystectomy	Model had accuracy of 91.1%
	Hashimoto et al. 2019 (18)	Retrospective cohort	Single institution, *n* = 88	Automated surgical phase recognition during laparoscopic sleeve gastrectomy	Model had accuracy of 85.6%
	Ramesh et al. 2021 (19)	Retrospective cohort	Single institution, *n* = 40	Automated surgical phase recognition during laparoscopic Roux-en-Y gastric bypass	Model had accuracy of 91.2%
	Hatib et al. 2018 (20)	Retrospective cohort	Multicentre, *n* = 1,334	“Hypotension Prediction Index” model to predict hypotension five minutes prior to true drop in blood pressure	Model had sensitivity and specificity of 92% and 92% with AUROC of 0.97
Postoperative	Stam et al. 2022 (21)	Systematic review	15 studies	Predict postoperative complications, including surgical site infection, postoperative anastomotic leakage, pulmonary complications, postoperative pancreatic fistula, 30 and 90-day readmissions, and mortality in colorectal, bariatric, gastric and hepatobiliary surgery amongst others.	Models’ AUROC ranged from 0.50 to 0.96 with majority of models outperforming conventional methods. Performance improved with inclusion of both pre- and intraoperative data
	Al Abbas et al. 2024 (22)	Retrospective cohort	Single institution, *n* = 4,581	Validate Grantcharov's “Operating Room Black Box” which uses computer vision and ML to identify adherence and quality of surgical safety checklists, intraoperative errors and events, surgeons’ technical skills, and environmental distractions (23)	Higher scores from the ML model correlated with postoperative outcomes, in lower mortality and decreased length of stay
	Hayashi et al. 2022 (24)	Retrospective cohort	Single institution, *n* = 524	Predict recurrence patterns of pancreatic cancer after upfront pancreatic surgery based on ML-based histological analysis	Variations in gland formation size and type of atypia predicted non-recurrence (AUROC 1.000), liver (AUROC 1.000) and lung (AUC 0.861) metastasis.
	St. John et al. 2024 (25)	Prospective cohort	Multicentric, *n* = 903	Validation of “LapBot Safe Chole,” an educational mobile game that uses Madani et al's GoNoGoNet model to provide users with real-time assessment on their choice of dissection of safe or dangerous zones of the hepatocystic triangle during laparoscopic cholecystectomy (10, 26)	Average scores were significantly positively associated with players’ case volume and training level. However, averages scores and confidence levels significantly decreased with increasing game difficulty levels.
	Lavanchy et al. 2021 (27)	Retrospective cohort	Single institution, *n* = 242	Automated evaluation of surgical skill during laparoscopic cholecystectomy	ML model predicted good or poor surgical skills with 87% accuracy and skill level with 70% accuracy
	Kitaguchi et al. 2021 (28)	Retrospective cohort	National database, *n* = 74	Automated evaluation of surgical skill during laparoscopic colorectal surgeries	ML model had accuracy of 75.0%

n, sample size; ML, machine learning; AUROC, area under the receiver operating characteristic curve.

### Preoperative applications

Prior to a patient's operation, numerous complex factors are deliberated by surgeons to deliver safe, quality and evidence-based care, in an efficient manner. Information such as indication for and timing of surgery, risk calculation, prognostication, time and resources required are some of these factors. Use of ML has the potential to more efficiently and effectively process the data required to advise about these factors.

Henn et al. evaluated the value of ML on guiding clinical decision-making to proceed with abdominal surgery, by conducting a systematic review of 47 articles with mean number of 55,843 patients (3). Surgical domains included the breadth of visceral general surgery including colorectal, surgical oncology, bariatric, and hepatobiliary surgery. Studies looked at predicting risk or benefit of procedures using ML vs. conventional decision-making. Standard measures of clinical decision making such as scores and tests, logistic regression, expert opinion and Cox regression were used to compare ML to traditional decision-making. 97.8% of the studies demonstrated ML to be superior to conventional methods in guiding clinical decision-making to offer surgery. They suggest that ML can be used to offer more personalized care, decrease costs by targeting high risk patients for prehabilitation and focused perioperative care, and that area under the receiver operating characteristic (AUROC) be used for ML model evaluation.

Another application of ML in the preoperative setting is prognostication. Chongo et al. studied ML use for prognostication of liver transplantation in a systematic review (4). 23 articles were included in which the ML models, using pre-transplant data, outperformed traditional scoring systems such as the Model for End-stage Liver Disease (MELD) and Child-Turcotte-Pugh scores consistently. Primary outcomes were mortality and post-transplant complications, in which AUROC demonstrated ML models’ excellent predictive performance (AUROC 0.9-1) for both short and long term outcomes. They conclude with highlighting the potential of ML models to optimize organ allocation, improve patient outcomes and decrease healthcare costs.

Furthermore, Zhao et al. used ML to predict case duration during robot assisted surgery (5). They performed a retrospective cohort analysis of 500 elective robot assisted surgeries of primarily abdominal visceral procedures at a single institution. 28 variables were selected for model building including patient age, obesity, tumor location, time of day, and surgeon postgraduate year, amongst others. Primary outcome was scheduled case duration as predicted by the ML model vs. the conventional system. All ML models including multivariable linear regression, ridge regression, lasso regression, random forest, boosted regression tree, and neural network decreased the average root-mean-squared error when compared to the baseline model. ML predicted durations were accurate 51.7% of the time compared to the traditional system's 34.9%. They highlight that ML can improve the accuracy of robot assisted case length predictions, to help improve utilization of scarce resources.

Li et al. explored ML in the preoperative setting by developing models for predicting surgical difficulty of laparoscopic rectal cancer resection (6). Surgical difficulty was scored based on duration of surgery, conversion to open procedure, postoperative stay >14 days, visceral fat area, body surface area and morbidities. 186 patients were included from a single centre institution. Four ML models were developed and validated, utilizing support vector machine, random forest, logistic regression, and decision tree. All models showed high performance using AUROC (0.9-1). Thus, the authors propose ML models can assist surgeons evaluate surgical difficulty preoperatively and accordingly make treatment decisions and resource allocations.

Cao et al. compared supervised ML models to predict severe postoperative bariatric surgery complications (7) based on preoperative factors. The ML algorithms were trained using 37,811 bariatric surgery patients from the Scandinavian Obesity Surgery Registry from 2010 to 2014, and validated on 6,250 patients in 2015. Preoperative data points such as age, BMI, year of operation, HbA1c, and obesity-related comorbidities were included in the ML models. 29 ML algorithms were studied including deep learning neural network, k-nearest neighbor, support vector machine, random forest, and adaptive boosting logistic regression to name a few. Synthetic minority oversampling technique was used to tackle the imbalanced data as only 3.2% of patients experienced severe postoperative bariatric surgery complications. Performance of the ML models were assessed using accuracy, sensitivity, specificity, and AUROC. Most of the ML algorithms demonstrated high accuracy (>90%) and specificity (>90%) but sensitivity (0%–75%) and AUROC (around 0.5) were poor for all models. They postulate this to the low incidence of postoperative bariatric surgery severe complications. They also note that studies that include information following the preoperative setting, such as intraoperative complications, show improved model performance. However, this is not useful for utilizing ML for preoperative risk calculations. Therefore, while potential is shown for the use of ML models in the preoperative setting in predicting severe postoperative complications, it is not clinically beneficial yet.

Lastly, ML has also shown potential in guiding appropriate timing of surgery. Lan et al. developed a ML model to predict timing of surgical intervention for infected necrotizing pancreatitis (8). A retrospective analysis was conducted on 223 patients in a single centre hospital. The ML models were logistic regression, support vector machine and random forest. Generative adversarial networks were used to generate simulated samples to overcome the small sample size. Compared to traditional models, the ML models better predicted surgical timing, as well as identified factors associated with surgical timing, and postoperative survival for infected necrotizing pancreatitis. They conclude stating that ML can provide good references for surgeons to make personalized surgical plans for infected necrotizing pancreatitis patients.

### Intraoperative applications

Along with providing valuable and personalized data in the preoperative setting, ML has also been demonstrated to have application in the intraoperative setting, which is particularly significant as the source of most adverse events for surgical patients can be traced back to intraoperative events (10). Areas that have shown promise are visual annotation of the surgical field, automated surgical phase recognition and prediction of intraoperative patient decompensation. This information can provide the surgical team with important information to optimize procedures, surgical training and ultimately provide more effective and safe care to patients.

Madani et al. created an AI model, GoNoGoNet, to visually display safe “Go” and dangerous “No Go” zones during laparoscopic cholecystectomy. This was generated using deep neural networks, a subset of AI algorithms that use machine learning (10). Laplante et al. validated GoNoGoNet with expert high volume surgeons from the Society of American Gastrointestinal and Endoscopic Surgeons’ (SAGES) Safe Cholecystectomy Task Force (9). They found the AI model to have 92% mean accuracy, 97% specificity, and 70% positive predictive value for safe zones and 92% mean accuracy, 80% sensitivity, and 95% negative predictive value for dangerous zones. Deep neural network modeling has also been used by Mascagni et al. who created a model, DeepCVS, to identify the critical view of safety in laparoscopic cholecystectomy with a mean accuracy of 71.4% (11). Another intraoperative use of machine learning in laparoscopic cholecystectomy is warning surgeons about adequate clipper tip visibility prior to clipping the cystic duct and artery. This model, ClipAssistNet, was also generated using deep neural networking by Aspart et al, which had a AUROC of 0.9107, 66.15% speciﬁcity and 95% sensitivity (12). Thus, these models may eventually be implemented for real-time intraoperative guidance to decrease the risk of bile duct injury during laparoscopic cholecystectomy.

Moreover, Garrow et al. assessed ML models for automated surgical phase recognition through a systematic review with 35 articles including laparoscopic cholecystectomy, sleeve gastrectomy, and colorectal surgery (13). The most common ML models used were Hidden Markov Model and Artificial Neural Network. Accuracy of the models ranged from 68.8% to 96.3%. Despite the majority of studies focusing on laparoscopic cholecystectomy, there was significant heterogeneity in the number of phases and definitions of phases, along with ML models used, precluding accurate comparison of studies. Interestingly, complexity of models and those used in more recent studies did not affect accuracy. Takeuchi et al. similarly looked at surgical phase recognition of laparoscopic inguinal hernia repair using deep learning models, displaying accuracy of 88.1% and 85.8% in unilateral and bilateral repairs, respectively (14). Kitaguchi and his team specifically studied automated phase recognition in laparoscopic sigmoidectomy and transanal total mesorectal excision using deep learning models with accuracies of 91.9% and 93.2%, respectively (15, 16). Cheng et al. likewise explored surgical phase recognition of laparoscopic cholecystectomy using deep learning models, and showed accuracy of 91.1% (17). Bariatric surgery automatic surgical phase recognition has also been studied. Hashimoto et al.'s deep neural based model showed 85.6% accuracy in laparoscopic sleeve gastrectomy (18). Ramesh et al. used multitask multi-stage temporal convolutional networks to automate phase recognition in laparoscopic Roux-en-Y gastric bypass with accuracy up to 91.2% (19). These studies conclude that automated surgical phase recognition is an emerging field and recommend data collection be compatible for ML analysis and standardized surgical phase definitions.

Another use of ML for intraoperative application is prediction of intraoperative decompensation. Intraoperative hypotension is associated with postoperative complications including higher mortality, acute kidney injury and myocardial infarction (29–33). Consequently, Hatib et al. developed an algorithm, the Hypotension Prediction Index, using machine learning to predict hypotension five minutes prior to actual drop in blood pressure. This was created using arterial waveform data of 1,344 patients, with a sensitivity and specificity of 92% and 92% with AUROC of 0.97 (20). The algorithm was validated in a randomized control trial along with a treatment protocol by Wijnberge et al. (34). Gastrointestinal, pancreas, esophagus, and gynecology procedures were included. They demonstrated a median of 16.7 min less of intraoperative hypotension for patients in the intervention group that used the Hypotension Prediction Index and treatment protocol compared to the control group. Therefore, this ML based model for prediction and prevention of intraoperative hypotension has the potential to decrease postoperative complications.

### Postoperative applications

Lastly, ML has been studied the most in the postoperative environment. Areas of interest are prediction of postoperative complications, recurrence patterns of malignancy, surgical education and automated surgical skill assessment. These applications have the prospect of providing patients with prevention and earlier management of complications, enhanced treatment after oncologic surgeries, and improved training and feedback for surgical trainees.

Numerous prediction models using ML have been developed for postoperative complications, particularly in the last few years (2). The range of visceral surgery including colorectal, bariatric, gastric and hepatobiliary has been studied. Surgical site infection, postoperative anastomotic leakage, pulmonary complications, postoperative pancreatic fistula, 30 and 90-day readmissions, and mortality are some of the complications that have been analyzed (1, 2, 21, 35–39). ML generally outperformed conventional logistic regression models (2). Performance can be improved by inclusion of both pre- and intraoperative data, instead of just one, along with both structured and unstructured data. One such intraoperative platform is Grantcharov's Operating Room Black Box platform which uses multimodal audiovisual data that is analyzed through computer vision and machine learning to identify adherence and quality of surgical safety checklists, intraoperative errors and events, surgeons’ technical skills, and environmental distractions (23). The ML-driven analysis from this platform has been demonstrated to correlate with postoperative outcomes. For example, Al Abbas et al. assessed surgical teams’ quality of surgical safety checklist performance, based on the scores output by the Operating Room Black Box, to postoperative outcomes (22). Higher scoring teams’ patients correlated with lower mortality and decreased length of stay. Therefore, these ML-based prediction models can foster improved and individualized management of patients’ complications in the postoperative period.

Hayashi et al. assessed a different aspect of postoperative care, in predicting recurrence patterns of pancreatic cancer after upfront pancreatic surgery (24). They presented a retrospective, single-centre study, with 524 patients in which histology-based supervised ML was used to predict recurrence patterns of pancreatic cancer. Variations in gland formation size and type of atypia predicted non-recurrence (AUROC 1.000), liver (AUROC 1.000) and lung (AUROC 0.861) metastasis. This information may lead to earlier, personalized chemotherapy for these patients.

ML has also been used to promote surgical education as demonstrated by Noroozi et al.'s LapBot Safe Chole, an educational mobile game that uses AI to provide users with real-time assessment on their choice of dissection of safe or dangerous zones of the hepatocystic triangle during laparoscopic cholecystectomy (26). The application uses Madani et al.'s deep neural network based GoNoGoNet model to provide feedback (10). St John et al. validated the study with 903 participants from 64 countries (25). Average scores were significantly positively associated with players’ case volume and training level. Meanwhile, averages scores and confidence levels significantly decreased with increasing game difficulty levels. This suggests that surgical education games can be an effective adjunct tool to offer practice and coaching for trainees.

Another potential of ML postoperatively is automated evaluation of surgical skill, which Lavanchy et al. assessed with laparoscopic cholecystectomy (27). 242 videos were used for annotation in the study. They developed a three-stage ML model to assess surgeon ability, based on surgical instrument handling. The premise being more skilled surgeons handle instruments in a focused area while less skilled surgeons have slow, trembling motions with repetitive direction changes and greater areas of movement. The three stages are instrument detection and localization, tracking motion, and skill prediction based on calculated motion metrics. Prediction skills ratings were correlated with expert ratings. The ML model was able to predict good or poor surgical skills with 87% accuracy and skill level with 70% accuracy. Similarly, Kitaguchi et al. also developed an automated surgical skill assessment tool for laparoscopic colorectal surgeries. They used 3-dimensional convolutional neural networking to create the model which demonstrated mean accuracy of 75.0% (28). The authors of these studies advocate that with a larger training database and model refinement, an improved automated surgical skill evaluation is possible to ultimately help provide continuous objective feedback on surgical skills for surgeons.

### Limitations and challenges going forward

Despite the scale of emerging data, there remains limitations and challenges in clinical implementation of ML in visceral surgery. The majority of this review's studies’ ML models were trained and validated internally with local institutional or organizational datasets as opposed to being validated externally. This can lead to potential biases and generalizability concerns to other patient populations. As well, most of the studies are observational (see Table 1), and thus, more statistically robust designs such as randomized control trials can be explored to validate the ML models in future studies. ML models in the studies that performed poorly were generally due to fewer modality sources of data and unstandardized and varying definitions of surgical steps or phases. As a result, there are less preoperative ML models compared to intraoperative and postoperative applications which can extract from more data points.

Quality and quantity of data affect ML performance. Vast amounts of data are required which may involve interdisciplinary regional, national, and international collaboration (40). The data is generally not standardized and requires immense time commitment to organize and annotate.

To help resolve this, the Global Surgical AI Collaborative has been established to offer surgeons access to large, international shared databases and an infrastructure to utilize this data (41). Federated learning, an encrypted and decentralized form of machine learning that allows data processing at remote physical locations to train local models which are then amalgamated into a final model. This increases data privacy and the possibility of greater data accessibility. Thus, models can be created collaboratively with other centres, mitigating the immense data annotation workload often required to create a single centre based ML model. Another method of decreasing the demand of data annotation is using coarser labels in which the content of a video sequence is categorized or qualitatively described instead of segmenting each frame (42). This method of labelling is less demanding, however may lead to less clinically relevant applications. Coarse labelling is suited for tasks such as navigation to specific points of videos as well as education by describing the contents of videos.

Secondly, appropriate resources and infrastructure to implement ML are necessary. Modifications to current electronic medical record systems are required to allow safe and real-time interaction between patient files and machine learning models (2). The ML models must also be trained and validated in the proposed healthcare facilities of installation to ensure accurate performance for that specific hospital. As well, incentivizing surgeons that participate in providing and annotating videos to standardized databases for AI video-based assessment, with compensation such as continuing medical education (CME) points, may increase the available data pool and improve ML models’ performances.

Lastly, ethical considerations and acceptance by patients and the surgical team are another barrier in the application of ML. To help address the ethical issues of using vast patient datasets, de Almeida et al. published a review and developed a framework for AI regulation, from legislation to research and development, with 21 guidelines (43). Moreover, in a systematic review of public perception on AI, patients generally viewed AI in positive light but still preferred to receive healthcare with human physician supervision over AI (44). Greater acceptance of AI was found if patients were given a choice between AI and provider, AI was applied in a low risk setting, AI was proven to be more accurate than providers, if physicians recommended AI, and if AI matched societal and cultural norms. While the evidence of ML in surgery is growing, surgeons are also weary of ML implementation without institution-specific validated models. It's important for surgeons to understand how ML works, help develop it, and then, push for its clinical application.

In summary, there is abundant potential of ML application in visceral surgery in the preoperative, intraoperative, and postoperative settings with the benefit of safer, more effective and higher quality patient care. Some challenges have created a gap between research findings and clinical implementation of ML which future studies should further address.
